# A transdisciplinary approach to nuclear waste management: Opening research with a Citizens’ Working Group

**DOI:** 10.1007/s13280-025-02214-9

**Published:** 2025-07-16

**Authors:** Roman Seidl, Cord Drögemüller, Pius Krütli, Clemens Walther

**Affiliations:** 1 Institute of Radioecology and Radiation Protection, Leibniz University Hannover, Hannover, Germany; 2 Justus Liebig University Giessen, Giessen, Germany; 3 TdLab, ETH Zürich, Zurich, Switzerland; 4 Risk Dialogue Foundation, Zurich, Switzerland

**Keywords:** Nuclear waste management, Science with society, Transdisciplinarity

## Abstract

Science often tackles sustainability challenges by not only addressing them (inter-)disciplinary but additionally engaging in societal aspects through transdisciplinary research. Siting a deep-geological repository is a technical and societal challenge. In this study, we present a unique approach that entailed a 4 year collaboration between our research team and a Citizen’s Working Group (CWG) on nuclear waste management in Germany. We explored whether and how knowledge could be effectively transferred among researchers and the CWG, as well as how mutual trust could be fostered and maintained. Seventeen ordinary citizens across Germany were selected via a multistep process, including an online survey and interviews. Their contributions provided valuable empirical insights and informed interdisciplinary research questions. We describe this new and challenging process with its multiple methods (surveys, workshops, observations, and interviews) and reflect on our experiences based on a rich set of empirical data. Finally, we discuss the potential for generalization and application of this approach to other topics.

**Supplementary Information:**

The online version contains supplementary material available at 10.1007/s13280-025-02214-9.

## Introduction

In this paper, we focus on a particular approach of involving citizens in research processes that, to our knowledge, has not yet been described in the literature. With this novel form of cooperation, we aimed to test its feasibility and to investigate the conditions for maintaining trust in researchers. This process was all the more challenging in a project with a current focus on the management of high-level nuclear waste (HLW) in Germany, including the ongoing site-selection procedure (Schafmeister 2023). While this subject has historically sparked intense debates and conflicts (Hocke and Renn 2009), our attention centered on exploring the collaborative potential between researchers and citizens holding middle-of-the-road views or being undecided on the topic. Those opinions could be described as ambivalent or indifferent and can be distinguished from other groups’ more pointed positions (Thompson et al. 1995; Seidl et al. 2013). Individuals with such ambivalent views may typically seek a middle ground, balance, and compromise. However, how to find and recruit such “silent” citizens presents a challenge. A recruitment or selection approach “designed to promote inclusion, paradoxically, does not adequately accommodate this emerging moderate group through the electoral process and existing representative institutions” (Pow 2021, p. 7). Therefore, those whose voices are louder than those of the public’s middle faction are often recruited into these groups. This effect had to be avoided in our study. We can refer here to the Modern2020 Project (Meyermans et al. 2019, p. 46) where a small group of engaged local community representatives consistently participated in project meetings and workshops that were conducted at the European level. Social scientists facilitated workshops or the so-called home engagement sessions in the communities of interested public stakeholders to address their concerns and opinions regarding the monitoring of nuclear waste repositories.

Actors in nuclear waste management and responsible politicians often mention that trust is an important resource and that it is necessary to gain public trust (Di Nucci et al. 2021). Trust may indeed be a prerequisite for fruitful debate in the first place, a basis for listening to the scientific findings or value statements of others. However, healthy mistrust (Lehtonen et al. 2022) may be positively perceived as civic vigilance (Sperber et al. 2010; Hendriks et al. 2016) and can also serve an important control function. In Germany, the current process is indeed under scrutiny by critical groups, as it was in the past (Di Nucci and Brunnengräber 2023).

Moreover, some scholars distinguish between social trust (usually between individuals, based on similar values, McKnight and Chervany 1996; Siegrist et al. 2000) and experiential confidence (in the ability of actors to perform their tasks, Earle 2010; and already by Luhmann 2017 [1979].

This distinction makes sense in the case of confidence in science or public administration (Metlay 2013) and social trust in scientists as individuals, which both played a role in our cooperation with average citizens.

Generally, engaging citizens in transdisciplinary research processes is an established approach used to help solve complex sustainability challenges. The reasons may differ from project to project depending on the intention, for instance including legitimacy, substantive issues, or normative concerns (Fiorino 1990; Krütli et al. 2010; Pohl et al. 2017). Furthermore, laypeople can contribute to science through citizen science (Criscuolo et al. 2023), which includes collecting data or posing research questions. They can also offer support through co-produced knowledge in transdisciplinary research projects (Schneider et al. 2019) or Living Labs (Bergmann et al. 2021). In these cases, a scientific agent, such as a research group, often leads the process and invites others to participate (Krütli et al. 2010).

Chambers et al. (2021, p. 984) point out two primary motivations driving co-production: the desire to address existing problems more effectively and the intention to reframe problems. Similar motivations underlie our *particular* approach, going back to the idea of “post-normal science” (Funtowicz and Ravetz 1993; Gibbons et al. 1994). Post-normal science proposes that in complex and uncertain situations, traditional scientific approaches may need to be supplemented by more inclusive and collaborative strategies that involve a wider range of stakeholders, including various publics. The recruitment of an extended peer group should help researchers build bridges, not only across disciplines, but also between science and the general public and practice (in the sense of science with society, cf. Seidl, et al. 2013). These “extended peer communities […] also possess, or create, their own ‘extended facts’. These may include craft wisdom and community knowledge of places and their histories, as well as anecdotal evidence, neighborhood surveys, investigative journalism and leaked documents” (Guimaraes Pereira and Funtowicz 2005, p. 75). In our project, the intended strategy was to engage citizens, who would not necessarily feel affected by the current site-selection procedure, in working together with researchers. Our main aim was to explore how mutual trust might develop over time. The goals and conditions of the work of our Citizens’ Working Group (CWG) were to complement the project for four years, attend workshops at least twice a year, read instructions, and learn the basics of nuclear waste management. They would receive a small allowance as compensation for their expenses. The CWG contributed by changing or supplementing our research team’s perspectives and posing challenging questions from the citizens’ viewpoints. For instance, researchers learned that the CWG is interested in monitoring technologies but also very much in the network of information and communication and ultimately in the decision-making process resulting from monitoring outcomes.

From the perspective of planning the recruitment of CWG members in 2019, the whole endeavor was a black box. It was difficult for our research team to anticipate what to expect from such a group. The project researchers’ previous experiences with groups from the public had not always been friendly, and it was expected that this topic could easily become quite emotional, and the discussion atmosphere might be problematic. Therefore, the goal was not to assemble a specifically tame group but at least to avoid group members who expressed unwillingness to discuss issues in a solution-oriented way. We therefore developed a three-stage selection process based on scientific concepts.

During the recruitment process and throughout our work with the CWG, we applied various methods, such as quantitative surveys, including open-ended questions and particular qualitative approaches (Caggiano and Weber 2023). Transdisciplinary research methods were also employed in the workshops.^1^

In the next section, we describe the aim and the process of the recruitment, including the selection of the CWG members. First, we would like to draw attention to the challenges in recruiting suitable participants for such a collaboration. Several reasons may lead to low resonance among the public (see also Warren, 2021 for his view on a fair selection of citizens). Concerning citizens’ recruitment for transdisciplinary research projects, to overcome its challenges, such as lack of awareness of the problem at hand, time constraints for attendance, lack of trust in authorities/project partners, and perceived lack of influence, scholars recommend certain *design elements* (Greiner et al. 2014; OECD 2022), “such as the random selection of participants, the payment of an allowance, and the role of facilitators in managing the process” (Voß and Amelung 2016, p. 752).

Furthermore, several selection criteria for our CWG were formulated upfront. One important requirement was that the members should not be experts or stakeholders in the field of final disposal of nuclear waste to avoid being stuck in the old opposition discourse or fundamental concerns about technical details. Technical debates are important but were beyond the project’s scope; however, there are formats in the site-selection procedure, such as “online consultation facilities, the Status Conference on Repository, workshops for youth participation, the citizens conference or digital dialog services” (Federal Office for the Safety of Nuclear Waste Management [BASE], 2025) designed to fulfill the inclusion purpose. Additionally, sociopsychological criteria (e.g., ability to work in a team) as well as balanced ratios with respect to sex, education, and age were taken into account. We suggested 15 ± 2 group members as a target number, small enough to manage and sufficiently large to deal with dropouts and obtain a variety of opinions.

As our work had practical and scientific components, we had the following research questions: Whether and how could knowledge be effectively transferred among researchers and the CWG? How could mutual trust be fostered and maintained? Another objective of the paper is to describe in detail the methodology employed in planning and implementing the transdisciplinary exercise. We also address the difficulties and opportunities that the participants faced during the exercise, thereby providing guidance for others who might wish to undertake similar exercises.

## Materials And Methods

### Screening process

In this section, we detail the screening process to illustrate the required effort and document the procedure for further use by scholars. We started a structured recruitment process by conducting a representative survey on trust in various agents (responsible authorities, science, media, etc.) and HLW management in Germany with approximately 5000 respondents (Seidl et al. 2022). At the end of the survey, we asked the participants whether they were interested in joining a CWG as part of the project, which was briefly described. Those who indicated interest (*N* = 703) were sent a link to the second short questionnaire (see online Appendix S1: Survey on CWG Recruitment). This screening survey comprised operationalizations of various concepts that appeared relevant. The analysis of this survey with 181 respondents revealed 49 potential candidates. Some (*N* = 28) were interviewed (via the video communication software Zoom) to obtain a better picture of these individuals and finally to select the required number of CWG members. These steps, the second survey, and personal interviews are described in more detail below, Sect. “Overview of the screening survey” and following. Further sections explain the lessons learned from that collaboration.

### Overview of the Screening Survey

We asked the respondents about their levels of experience with citizens’ initiatives or charitable/voluntary work to determine whether they had experience with the work that such activities would entail. Although it was not a requirement, it would be good to know that information.

Moreover, the participants should be interested in social decision-making processes and technical problems. We wanted to see whether there was a general interest in current political events and technical issues. We used the concept of technocratic versus social world views according to Buss and Craik (1983); for the German version, see Siegrist (1999). This instrument asks respondents to rate items such as “Hard-headed rationality should be the basis of decision-making in our society.” Moreover, a scale (Neyer et al. (2016) measured technology commitment, that is, attitudes toward and use of modern technology.

The ability to work in a group was considered an essential characteristic of the prospective CWG members. According to Seelheim and Witte (2007, p. 81), teamwork skills comprise communication skills and other abilities (to interact, cooperate, deal with conflict, integrate, reach consensus, and be sociable). We assessed these skills roughly by applying instruments from the literature that seemed short enough and appropriate (Rohrmann 1978; Porst et al. 2008). For instance, the participants were asked how often they were involved in “giving speeches or presentations to at least five people” and other activities. This was an assessment of how they might be able to handle discussions with one another or with researchers and stakeholders.

General social skills were measured according to the survey suggestions by Lang (2008). For example, the respondents were asked to what extent the statements about their abilities to cooperate, deal with conflict, and communicate applied to them personally, such as “express criticism constructively,” “seek a compromise in the event of differences of opinion,” and others.

We also applied four items from the 21-item version of Schwartz’s *Portraits Value Questionnaire* (Schwartz 2012). The German version (Schmidt et al. (2007) was used to assess the importance of certain values, for example (female version): “It is important to her to listen to people who are different from her. Even when she disagrees with them, she still wants to understand them.” Because of anonymity in the survey, we did not know whether a respondent was male or female, so we adapted the items to the first-person perspective.

We also asked whether the participants would generally describe themselves as very impatient or very patient, as defined by Prüfer and Porst (2010). This personal trait seemed important to avoid frustration since not all participants in a discussion group would think and speak at the same speed.

Because the project would run for another four years at the time of the survey, we also assessed how long the participants could imagine working continuously in the CWG.

The final section’s open-ended question asked the respondents to write a few sentences about their personal motivation and goals for the CWG. One purpose was to learn more about individual intentions and motivations; another was to increase the participants’ effort and thus raise the bar (reduce the sample for easier selection).

## Results

### Results of the screening survey

The data from the 181 individuals who completed the questionnaire were analyzed statistically (items) and qualitatively (motivational texts). Cluster analysis (Aldenderfer and Blashfield 1978; Ward technique, sum of squares index; Jain et al. 1999) of the items on teamwork, values, and technical interest revealed a rational five-cluster solution. We checked the three-, four-, and five-cluster solutions, but the last one could be interpreted most easily. Clusters 1 (*N* = 30, mean age = 47 years), 2 (*N* = 25, 56 years), and 3 (*N* = 84, 47 years) showed interesting profiles regarding the items and scales used (i.e., high values for teamwork). The motivational texts were evaluated using the traffic light system. Two of the authors independently rated the texts and classified them as green, yellow, or red, depending on how useful or suitable they appeared for the intended task. Green meant “possibly suitable for the CWG.” The following are examples of statements rated under each category. Green: “Because I find it very interesting to be able to contribute my views on these difficult topics and then discuss various aspects together with others. Precisely because as a layperson you often don’t get the chance to express your rather uneducated opinion, this opportunity is a great chance for people like me to perhaps make a difference.” Orange: “I want to take part in discussions and make a difference, initiate thought processes, exchange ideas, discover new things, learn, broaden my horizons.” Red: “I would like to educate the masses about how much the disposal of radioactive waste actually costs and that we as taxpayers finance the whole thing, and very importantly: the polluter of this waste does not have to pay anything.”

As a result, 66 (rater 1) and 64 (rater 2) motivational texts were colored green. The two raters had ~ 75% overlap—that is, 49 motivational texts were rated similarly as green. Only these 49 cases were considered in the further procedure. As we applied these different methods to classify the respondents, interestingly, only Clusters 1 to 3 (found through statistical analysis) showed green motivational texts, based on independent qualitative rating. Clusters 4 (*N* = 16, 35 years) and 5 (*N* = 26, 42 years) offered no, bizarre, or only rudimentary texts rated as red or orange. Thus, the mixed methods cross-validated the results.

### Results of Interview

Since not all 49 individuals could be interviewed due to time and organizational constraints, an upper limit was set for the number of interviews. A selection of 30 individuals with “green” texts and “good profiles” had to be made. Sex, age, and educational background were also taken into account to achieve a balanced sample from which potential candidates could be selected. To ensure a similar procedure for the 30 semi-structured interviews to be held virtually via Zoom, an interview guide was created, consisting of the following parts:

- Personal background questions (5–7 min)
  o *The respondent should briefly introduce himself/herself.*
- General information on the topic of final disposal (5 min)
  o *The interviewer informed the respondent about the project and the tasks.*
- Questions from the candidates about the project and its progress (7–10 min)
  o *The respondent could ask any question about the project and the potential tasks.*
- The candidate’s questions concerning the project (5–7 min)
  o *What may be particularly positive conditions or, instead, no-go’s for good cooperation?*

The complete list of guidelines with subquestions is provided in online Appendix S2: Interview Guidelines. Two of the 30 planned interviews did not take place for technical reasons. The interviews with 28 individuals were usually conducted by three interviewers (sometimes by two) from June 18 to July 7, 2020. The individual impressions were compared and discussed among the interviewers. The candidates were rated independently on a scale of 1–5 on the following indicators: overall impression, ability to cooperate, ability to work in a team, communication style (during the interview), and special observations. The higher the score, the better the suitability for the project. The three interviewers discussed each case and selected 17 candidates (Table 1).

**Table 1:** Selected candidates for the CWG. The information relates to the time of recruitment. The last column shows who is still active in the CWG until the end of the project

Age	Sex	Education/Work	Currently still active in the CWG
20	F	Voluntary social year	Yes
22	M	Student	Yes
24	M	Student (dual course)	Yes
25	F	Writing dissertation (for doctor of dentistry)	No
37	F	Police press officer	Yes
37	M	Network technician (IT)	No
43	M	Legal assistant	No
44	F	Early retiree	Yes
44	M	Self-employed	Yes
47	F	Project manager	Yes
49	F	Art director (graphic designer)	Yes
50	F	Environmental technologist	Yes
57	F	Excavation technician	Yes
58	M	Warehouse worker	Yes
61	M	Application developer	No
65	F	Early pensioner	Yes
69	M	Pensioner	Yes

### Resulting CWG members

While not a representative sample of the German population, the chosen individuals comprised a diverse group of citizens representing different age, educational, and occupational segments, as well as gender parity and various geographic regions of origin. As Table 1 shows, the 17 selected individuals covered a wide range of educational backgrounds and professions, from student to early retiree, from graphic designer to excavation technician (experienced in archeological projects).

### Work with the CWG after selection

#### Kick-off meeting

The collaboration began with the first face-to-face meeting on September 26, 2020, in Hanover, Germany. Of the 17 selected citizens, 15 traveled from 7 German states, despite difficult COVID-19 conditions. The CWG members were asked about their motivations during the introduction round at the inaugural meeting, using moderation cards. Some of the motivations written down are as follows: “To learn new things, expand my knowledge, try things out,” “To create a safe future for all, active participation, exchange with those involved,” and “To see an important and necessary process from close up and, if necessary, contribute new aspects with my opinion and perspective.” During this meeting, the CWG members were also introduced to one another and to the scientists. Detailed information about the project and possible activities was given, and the CWG’s role was explained to the members. An important step was the constitution of the CWG in self-organization and the discussion on the “working basis document,” including the formal conditions of cooperation, financial rewards, and so on. Additionally, the conditions of collaboration were explained, for instance, regarding the CWG’s suggestions to the researchers. While the CWG could suggest research topics, it should not dictate specific questions. However, if the researchers chose not to follow the CWG’s suggestions, they must provide a reasonable explanation. Furthermore, two members were designated as group speakers (one female, one male), a role they assumed and maintained throughout the project’s duration.

Two male members left the process early for personal reasons. There was no indication that a lack of trust might have been the reason for their departure; instead, both cited professional problems (job change). Two female members became inactive after about two years. Their reasons could not be investigated because they did not respond when requested to participate in the final wrap-up interviews.

#### Observed workshop series

Table 2 provides an overview of the workshops in all work packages. It includes the kick-off meeting but excludes the introductory workshop (March 2021) on the transdisciplinary approach and specific methods, exemplified by the emancipatory boundary critique methodology (Pohl 2020). Over the 4 year period of project work, we held 6 workshops on 2 topics: (1) host rock and retrievability and (2) near-field monitoring of the waste in a deep-geological repository (TRUST). Five additional workshops were held, with another subproject on the safety case (SAFE). In the remainder of this paper, we report the work in the work package TRUST. Workshop 6 (under TRUST) was more self-organized with those CWG members who were interested in writing a citizen’s opinion report on waste retrieval. It was not observed in the same way as Workshops 1–5.

**Table 2:** Overview of the workshops held with the CWG

Workshop	Topic	No. of CWG members	Date/s	Format
0 All	Kick-off meeting	15	Sept. 26, 2020	Face-to-face
TRUST 1	Trust—the psychological perspective Trust and monitoring	14	March 12/13, 2021	Online
TRUST 2	Retrieval, uncertainties, and trust	11	Sept. 24, 2021	Online
SAFE 1	The safety case	8	Sept. 25, 2021	Online
SAFE 2	The FEP catalog (*features, events, processes*)	5	Dec. 11/12, 2021	Online
TRUST 3	Monitoring concepts	7	May 6, 2022	Face-to-face
SAFE 3	Scenarios and expected developments	6	May 7, 2022	Face-to-face
TRUST 4	Retrievability and host rock	7	Oct. 21/22, 2022	Face-to-face
TRUST 5	Monitoring: transparency, decision, procedure	10	May 6, 2023	Online
SAFE 4	Radiation exposure and effective dose	10	June 2/3, 2023	Online
TRUST 6	Citizen’s opinion report	5	April 27, 2024	Face-to-face
SAFE 5	Effect of the repository in the underground	5	April 26, 2024	Face-to-face

#### Observations of interactions

In our project, we conducted open and non-participatory observations (Vidich 1955; Jorgensen 2020); the CWG and the researchers being observed were aware of these. For some researchers, the situation may have been artificial in that they were not used to dealing with citizens (who comprised the CWG) in their scientific work. Similarly, for the CWG members, the setting was not their normal living or working environment. However, meetings/workshops were conducted in a relatively natural context (workshop setting, often in a hotel seminar room), not a laboratory at a psychological institute.

The interactions between the scientists and the CWG members were systematically observed using a self-developed observation matrix (see online Appendix S3: Observation Matrix). We paid particular attention to trust and its possible development over the course of the collaboration. To our knowledge, since the literature did not provide an adequate survey instrument for the observation of trust, a new instrument was developed based on other instruments (Bales 1988) and thematic literature. A distinction was made among different types of communication, such as a question, a question for a specific person, input, a (longer) presentation, and others. Additionally, some verbal indicators that could indicate trust or mistrust (e.g., control questions, showing interest, approval) were developed.

To record any dissatisfaction and changes in attitude toward content-related issues as a result of the collaboration, online surveys were sent out to the CWG after each workshop (see also online Appendix S4: Survey on attitudes after workshop [examples]). Attitudes toward repository issues were also surveyed regularly, particularly regarding retrievability and monitoring.

#### Wrap-up interviews

We conducted final interviews (via Zoom) with 12 members of the CWG in April and May 2024 to wrap up their views on the collaboration and to clarify open questions (see online Appendix S5: Interview guideline for the CWG—wrap-up interviews). The key topics were potential changes in the members’ motivation to join and to stay with the CWG, respectively; the same was the case for trust. Moreover, the expected and the actual roles of a CWG member were examined. The interviews were recorded, and the audio files were automatically transcribed using Trint software. The transcripts had to be read and corrected to eliminate errors introduced by the software. For this paper, the transcripts were analyzed with MAXQDA. The results are captured in Table A1 in online Appendix S6: Summarized results from the wrap-up interviews. The complete dataset (in German) can be explored in an online repository^2^.

### The working process: observed and analyzed

The essential criteria for citizens’ evaluation of a trustworthy process are openness, transparency, and fairness—if an actor can demonstrate technical competence and high standards in these criteria, trust can be fostered and acceptance increased (International Atomic Energy Agency 2022, p. 17). We now discuss the results of our analysis concerning trust building (Sect. “Results on the research question on trust building and reflection on the process”) and knowledge (4.2).

#### Results on the research question on trust building and reflection on the processs

**Fig. 1:**
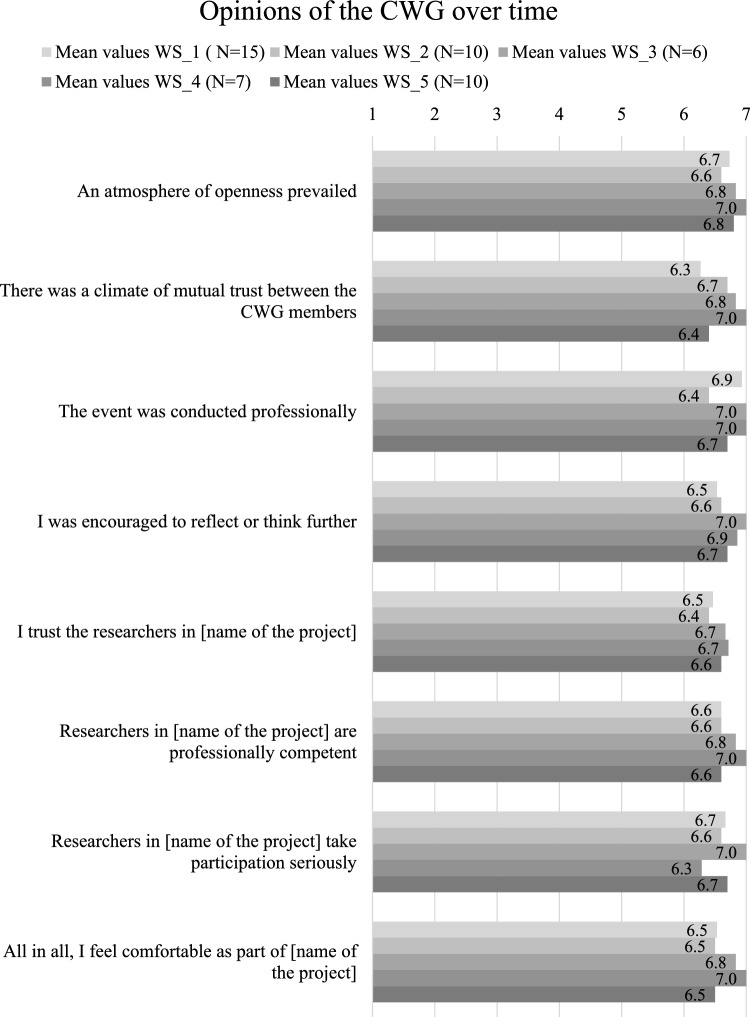
Trust values obtained from the regular follow-up surveys. *Notes* The response scale ranged from 1 = does not apply at all to 7 = applies fully. Note that the workshops were held in different configurations and did not always involve the same members of the CWG. The range of responses on the scale was 2; for each item, the min value was 5 and the max value was 7. (Color figure online)

Experience has shown that the CWG functions as such well, with its smart self-organization and generally positive atmosphere. No major conflicts would have led to a split in the CWG. Emerging disagreements have been addressed and resolved internally. It is accepted that there are different views on certain issues and procedures. The empirical results of the follow-up surveys, as depicted in Fig. 1, indicate overall satisfaction and a notable level of trust over the course of the workshops. The majority of the CWG members have stated that all relevant information is shared with them, emphasizing openness and transparency.

Moreover, the coding from the observation matrix reveals that mistrust and critical rejection of statements from researchers are generally rare, while factual talk (German: *sachlich*) is most prevalent. (Table 3 summarizes the frequency of each point over the course of the workshops and gives a rough impression of what kinds of interactions prevailed.) During some phases of the workshops, many questions were posed to the researchers, mainly because CWG members needed information and more context. Then, “control questions” also occurred, in the sense that some members wanted to dig deeper and challenge the researchers. Note that contributions such as “Contradiction/criticism” could also be directed toward other members of the CWG or among researchers.

**Table 3:** Categorized types of communication and indicators of trust/mistrust during five workshops

Type of communication	Sum per category	Indicators of trust/mistrust	Sum per indicators
Remark	223	Factual/objective	265
Answer	156	Critical	85
Discussion	129	Approval	35
Explanation	76	Show interest	34
Question to an individual	66	Offer/ask for help	29
Question to all	60	Contradiction/criticism	23
Input (e.g., technical)	60	Provocation/aggression	13
Presentation	54	Passing on/disclosing critical (confidential or insider) information	13
Moderation/Discussion lead	34	Control (question)	12
Query to an individual	7	Changing the subject	6
Query to all	2	Undermining expertise	5
		Seeking contact with certain people	5
		Rejection	0
		Concealment	0
		Distortion	0

To answer the research question, various measures and conditions can be identified as contributing to or favoring a trusting relationship between researchers and non-researchers. Table 4 systematically presents these framework conditions (i.e., enabling factors) throughout the course of the project. Our overview refers to the conditions that a researcher can influence by designing the collaboration and specific workshops. A distinction can be made between interpersonal and more formal or technical conditions, which can relate to trust building, on the one hand, and to knowledge generation and integration, on the other hand.

**Table 4:** Framework conditions for trusting cooperation. The abbreviations refer to explanations in the main text. *TI* trust interpersonal, *TFT* trust formal-technical, *KI* knowledge interpersonal, *KFT* knowledge formal-technical. Generally, the conditions are not related, except for TI4 and TFT2 and TI5 and TFT3 (indicated by arrows)

	Interpersonal		Formal-technical	
Trust	TI1	Being on first-name terms (closeness, eye level)	TFT1	Working basis document (security, transparency)
	TI2	Informal exchange, closeness, openness		Shared meals
	TI3	Conversations during breaks (informal exchanges, including those about private activities)		Shared meals
	TI4	Possibility to chat via one’s own WhatsApp group (self-organized) ↔	TFT2	WhatsApp group (technical chat solution)
	TI5	Regulars’ table/*Stammtisch* (self-organized) ↔	TFT3	Zoom account (technical solution for video calls)
Knowledge	KI1	Informal channel (content-related and organizational information, approachability)	KFT1	Cloud repository (technical solution for data exchange)
			KFT2	Zoom account (technical solution for exchanging content-related information, e.g., writing a book chapter)
			KFT3	Area on the project homepage (technical solution for providing information)
			KFT4	E-mail distribution list (technical solution for exchanging information)

The modalities and guidelines for future cooperation were set out in a joint document known as the working basis document (see Sect. “Kick-off meeting”; TFT1 in Table 3). In our opinion, this rather formal and technical step created transparency and security from the outset and thus laid the foundation for a trusting relationship. Two CWG members made explicit reference to this view during the wrap-up interviews, one stating that a solid foundation had been laid by discussing this document at the kick-off meeting.

The CWG’s requests for their own Zoom account (TFT3) and a workspace on a shared cloud (KFT1) were implemented immediately after the kick-off meeting. Such accommodation required the disclosure of private data, which was initially not welcomed by everyone. Professionalism and security were demonstrated through careful handling of the data. For example, an e-mail distribution list was created, not only for the subprojects, but also for the CWG, where the individual e-mail addresses were hidden. Technical tools facilitated early scheduling and timely distribution of information materials for workshop preparation, preventing frustration. Informal channels (KI1) lowered communication barriers between researchers and CWG members, enabling the spontaneous and direct exchange of content, organizational details, and thematic knowledge. Our observations showed that timely delivery of materials before workshops was an important topic.

As cooperation progressed, it became clear that even small actions could have huge effects. Extending the same kindness found in the CWG (TI1) to collaborating scientists enhanced their mutual trust. Worries about shifting from the German formal “*Sie*” to informal “*Du*” were proven unfounded as it helped break down barriers and build a closer community without sacrificing respect. Maintaining a courteous working environment throughout the project built trust and encouraged initially reticent participants to become more involved, asking questions and sharing opinions in larger groups.

According to our observations, other interpersonal conditions also had a trust-building effect. These included joint lunch and dinner (TI2) and exchanges outside the official work phases, such as in breakout sessions during the workshops (TI3) or informal “chatting out of the sewing box.” Other informal spaces were created through technological means (KFT1–KFT4). A virtual CWG regulars’ table (TI5) and the CWG WhatsApp group (TFT2) are worth mentioning since they allowed easy and fast direct communication and even fostered friendly personal contacts among some members. This channel was used for discussions around project/task-related content as well as private matters. The positive group feeling and cohesion among the CWG members were noted by five of them in connection with building trust. One member even described the group as a safe space. An overview of the most often mentioned aspects is shown in Table 5; more details can be found in online Appendix 6.

**Table 5:** Categories of trust-related statements (most frequently) mentioned during the wrap-up interviews (*N* = 12)

Category	Frequency	Example of a quote
Basic trust in science	9	There is simply a leap of faith because for me, this trust is simply there, that science acts neutrally and doesn’t pursue—I’ll call it its own interests—but simply generates knowledge on this objective, rational level. (Interview 01)
Openness, transparency, and honesty	9	… that you could be there after all and had the feeling that yes, you might not understand everything yet, but you can listen. It’s not a closed circle; you’re welcome there. And I think that strengthened the whole thing. (Interview 10)
Group cohesion/ safe space	7	So, I think if I had somehow not felt comfortable in the group or somehow not been taken seriously by the scientists and there had somehow been no trust, then I would have dropped out, too. Yes, because I think that’s very important somehow, especially when you’re expressing your opinion on something, that you can do it in a safe space like that. (Interview 04)
Getting to know one another and researchers	7	And by getting to know each other and working together, we have also experienced that we are taken seriously. … And that naturally strengthens the trust, also this mutual appreciation. … So it has changed a bit from the general public in my opinion, in my view, in relation to the people we work with. (Interview 08)
Eye level/respectful interaction	5	But overall, personal reference, listening to conversations at eye level, and responding to other arguments. That what is said is not simply dismissed but taken into account. I think these are crucial points that have strengthened and can strengthen trust in science. (Interview 07)

Professional planning and implementation of the respective events (result-oriented formats) also had a trust-building effect because the CWG members felt that they were being taken seriously and that the results were important to the researchers. During the wrap-up interviews, many CWG members reported things that could be coded under the “Openness, transparency, and honesty” category. The researchers were complimented for their openness and honesty, which fostered trust, according to the interviewees. The researchers demonstrated such traits by acknowledging their limitations in knowledge, addressing questions within and beyond the scope of the topic, and welcoming the participation of laypeople. These qualities were emphasized by seven members. Three individuals also mentioned the open interaction among the CWG members as a valuable aspect that strengthened trust. One member postulated that this practice benefited not only the CWG but also the younger researchers.

Furthermore, discussions without a precise formulation of the research questions to be addressed could quickly get out of hand, so that in the worst-case scenario, a lengthy conversation at the end would miss the point. In this situation, intervention and readjustment by the researchers were occasionally necessary and explicitly desired by the CWG.

The offer of virtual meetings exceeded expectations, with CWG members showing increased participation in online or hybrid-format workshops and project meetings (see Table 2). While face-to-face meetings had been welcomed since 2022 (after the COVID-19 pandemic), logistical challenges and time constraints associated with distant venues and travel remained significant hurdles. As one CWG member admitted during the wrap-up interview, for her as a somewhat shy person, online workshops helped through the formalized process of raising her (virtual) hand.

However, on-site exchange was still deemed indispensable for complementing digital interactions and leveraging the benefits of face-to-face meetings, such as shared dinners. This became evident in an informal survey we conducted via the WhatsApp group after we suspected a potential connection between the meeting format and attendance. Interestingly, in that survey, the CWG members preferred in-person meetings over online or hybrid types.

Our observations also revealed that working in smaller units, such as breakout groups in online sessions, supported more intensive exchanges. In larger discussion groups, not everyone would have the opportunity to speak, and some might hesitate to contribute. This was obvious at the start of the collaboration, especially during face-to-face meetings over the course of the project.

#### Results and reflections: Does more shared knowledge increase trust or mistrust?

In the initial years of the project, the CWG members accumulated substantial knowledge through active participation in workshops, working meetings, conferences, summer schools, and excursions, as well as through independent efforts such as targeted monitoring of reports and exploring media libraries. The exchange of knowledge, both from the scientists to the CWG and within the CWG, was further facilitated by the establishment of appropriate technical framework conditions, as mentioned earlier (see KFT1–KFT4 in Table 4, e.g., the Zoom account or the information area on the project homepage). Regarding the question of whether more shared knowledge increases trust or mistrust, regular follow-up surveys clearly indicated the former (cf. Figure 1). This result was also confirmed by the personal impressions of the scientists. The deepened knowledge of the complex repository problem (especially the work on uncertainties) clearly did not make the CWG members more distrustful of the researchers in the project. This quote from a CWG member during an observed workshop on the monitoring challenges and potential risks illustrates the development: “Of course, there are risks and uncertainties, but I don’t worry so much any longer. One becomes more professional.” The wrap-up interviews were rich sources of materials concerning the potential development of this trust. The CWG members agreed that over time, trust in science in general had been complemented by personal trust in us as researchers. They became aware that it was possible to deal with risks and uncertainties and to accept that not everything was known, but decisions still had to be made on critical issues. In two interviews, it was mentioned that with deeper insights and exchanges with researchers regarding the associated risks, the members became unsure of their own preliminary opinions and revised them in the course of the interactions.

Notably, the citizens gained a more profound understanding of the day-to-day research undertaken by the project’s scientists, discovering that not all experts share uniform perspectives but present diverse views and interpretations. It became crucial to embrace expert dissent without undermining trust in science overall. The coding of the observations (Table 3) and the results of the follow-up surveys show that trust in the scientists involved remains at a relatively high level.

#### Enabling factors—toward a framework of fruitful interaction

Generally, the scientific language had to be adapted to the level that the CWG members could understand. Particularly at the beginning, specific terms—known to the researchers specializing in the domain under study but not to their colleagues from another scientific discipline nor to the CWG members—had to be “translated” and explained. This didactic reduction of complexity helped the process in two ways. First, it showed the CWG members that the researchers took them seriously and did not shy away from the extra effort to be comprehensible. Second, simplifying without eliminating uncertainties helped the CWG members understand, if not fully comprehend, the issue at hand, which they considered important.

Moreover, the workshop design had to be adapted to tap into the knowledge, values, and attitudes of the citizens. What did not work was to simply pose the scientific research questions to the CWG. The researchers had to learn how to ask the questions needed to obtain the desired results. In the beginning, the researchers also tended to let the citizens discuss the issues freely, not to be too restrictive nor to steer the conversation to a particular topic. However, it turned out that the CWG members were most productive (also in terms of scientifically valuable outcomes) when the workshop design included prompting methods rather than a freewheeling discussion of a question. For example, in workshop TRUST 3, it proved advantageous to create a task where subgroups of three to four CWG members had to match several prepared terms/statements on a two-dimensional matrix. This practical application after a short discussion forced the members to produce observable results. In the follow-up survey at the completion of workshop TRUST 3, they stated that this type of work was also more satisfying for them since they could recognize their work as actually productive and relevant (e.g., regarding the question, “What would you like to see at the next workshop on monitoring?”, a respondent replied, “Tasks like the pinboard task: clearer task definition enables more targeted work.”).

Some participants also indicated in the wrap-up interviews that appreciative interaction (*N* = 2), meeting at eye level (*N* = 2), and the experience of being taken seriously (*N* = 2) were conducive to building and maintaining trust. Likewise, one person noted that a scientist’s visit to the regulars’ table (TI5 in Table 4), taking extra time to make a scientific contribution, was a source of trust. Therefore, some degrees of freedom in the design of the collaboration and particular workshops enabled trust and knowledge sharing.

## Discussion

Trust in experts is needed if one lacks special knowledge on a topic (Siegrist 2019). Trust is also a prerequisite for accepting risks and uncertainties in communication from experts (White et al. 2011; Eiser et al. 2012). In our project on high-level radioactive waste management, we aimed to involve citizens (comprising a CWG) who would not feel excessively affected by the current repository site-selection procedure in Germany and did not have a strong attitude toward nuclear waste but had different experiences with its special German history. As an “extended peer group,” these citizens collaborated with researchers (Funtowicz and Ravetz 1993).

One pivotal enabling factor was the selective recruitment of citizens. At the end of this process, 17 individuals were chosen as highly fitting. The CWG’s ability to function in day-to-day project work was demonstrated over the course of the project. The group displayed strong dedication and set itself apart by advocating for critical analysis and requesting involvement and information. The CWG members’ intention to contribute to a scientific undertaking was tangible.

In the field of climate science, Gaziulusoy et al. (2016, p. 57) identify three types of challenges for teams working in transdisciplinary projects. The third challenge concerning *teamwork* is of greatest interest for our topic: Teamwork challenges “stem from the requirement of collaboration of researchers from different expertise backgrounds and often from different academic institutions with each other and with non-academic stakeholders in ways to enable transdisciplinary knowledge generation.” We had an interdisciplinary research team, whose members ranged from social scientists to radioecology experts and geologists. Our team had to deal not only with proper communication among researchers but also with the CWG members. It was unclear from the outset whether trust would be developed and how it could be accomplished. Thus, our initial practical assumptions might have been biased toward the expectation of mistrust and the need to build trust.

A body of empirical evidence suggests that three dimensions—expertise, integrity, and benevolence—influence trust in scientific knowledge (Hendriks et al. 2016). As apparent during the course of the analysis, the CWG members exhibited an increasing degree of confidence in the researchers’ expertise, abilities, and skills. They were also convinced of the researchers’ general goodwill (i.e., benevolence), motivated by the desire to address the nuclear waste problem through scientific means. As often mentioned during the wrap-up interviews, trust evolved as a product of reliability cultivated through shared project involvement and the objective to contribute to the solution to the HLW problem. Additionally, the CWG perceived the researchers as having no hidden agenda, and their individual interests and were made explicit. For example, one research group showed a preference for salt (as a host rock) over clay. This favorable impression also included the researchers’ honesty in acknowledging uncertainties and the need for additional research.

Such self-criticism and transparency were well received by our group of citizens. By its very nature, science welcomes suspicion and vigilance, and it has self-control mechanisms to systematically check research results, such as the (far from perfect) peer-review system. While a certain amount of trust among scientists is necessary to build one's own research on the results of others, the process of "critical review" by peers can be seen as an institutionalized and systematic form of mistrust within the scientific community. Therefore, people might trust science for this reason as well: “Trust in Science does address a paradox, as Science has evolved as a means to question and readdress established ‘facts’. As such, the very idea of modern Science is to know the truth instead of just trusting what you are told” (Hendriks et al. 2016, p. 145).

The temporal dimension is a further cause for the two groups’ high level of mutual trust. Over four years of collaboration, the CWG and the researchers consistently experienced being able to rely on each other. The results indicated that the CWG started with considerable trust in science in general and, over time, considered the project researchers personally trustworthy due to their open-mindedness and ability to connect with the CWG members. Concerning the more differentiated terminology introduced above, the former, that is, trust as confidence in science, can be distinguished from the latter as (inter)personal trust. The abstract notion that science fulfills its role by producing facts that lead to informed trust (Bromme and Hendriks 2024) is then complemented by interpersonal trust. The latter is fostered by the citizens’ experience that the scientists whom they encounter during coffee breaks are also, in a way, ordinary people: “Lay people are more likely to trust and engage with science when they learn that researchers are human beings, fallible and conflicted” (Sandford 2020, p. 339).

The results of Tuler and Webler’s (2023) study indicate that four key perspectives may inform effective facilitation of community decision-making processes. These perspectives include: (1) accelerating the process of arriving at a consensus, (2) fostering acceptance through the cultivation of trust, (3) promoting inclusivity and transparency, and (4) establishing the legitimacy of the process and its outcomes. As explained earlier, we initially focused on the second perspective and the positive aspects of trust in science and researchers. However, there may also be some downsides. In public discussions or conference presentations, we have been asked whether the CWG is too trusting by just believing everything we say. Our data show that this is not the case. When confronted with this concern, the CWG members clearly stated that a trusting environment allowed them to question a professor’s proposal in the first place (i.e., showing healthy mistrust, although this term was not used). From a citizen’s perspective, researchers (especially renowned professors in a complex scientific field) are experts whom one does not often encounter. Some reluctance to really challenge them is understandable. Thus, the perspectives found by Tuler and Webler (2023) may not be independent dimensions, but (2) and (3) depend on each other, and (4) could be a result of proper participation.

Our analysis suggests that a central prerequisite for the success of transdisciplinary approaches is above all the openness of those involved in the project and thus the willingness to embrace new ideas and welcome experimentation. This receptive attitude also includes not smirking at or dismissing questions, remarks, or comments from citizens—even if they are unfounded from a scientific domain’s perspective—but making an effort to answer them seriously and within the scope of the given possibilities in the style of good scientific practice (Thompson 2009). At the societal level, a positive manifestation of mistrust can be civic vigilance, regarding, for example political influence on the publication of research results, the siting procedure’s fairness, or a premature focus on one of the three possible host rocks in Germany (Schafmeister 2023). Moreover, Germany may be a special case since many different cultures exist among the European nuclear countries. In their study on journalistic vigilance and the watchdog role of the press (serving society), Lehtonen et al. (2021) compare French and Finnish newspaper articles and conclude that there are societal differences due to “the Finnish trust‐based and the French mistrust‐based political cultures and traditions.”

Without delving too deeply into the theorization of trust, we could state that our experience with the CWG suggests a type of basic trust (personal or social) as a factor enabling vigilance, a kind of encouragement to question even those whom one trusts. The question of whether trust in institutions needs to be complemented by interpersonal trust cannot be answered here. Referring to a comprehensive book on trust and democracy, edited by Warren (1999), many contributors to that volume broadly agree that the core of trust indeed lies in the interpersonal sphere, and they question whether it makes sense to speak of trust/confidence in (political) institutions. Conceptual issues aside, it may be generally wrong to trust the government (without good reasons) or those in power because there have been examples of abuse of such power and instances of maladministration (Norris 2022). One might prefer a deliberative–democratic approach in which trust and deliberation are in a complementary relationship. Through an institutionalized principle of publicity, trust is supposed to alleviate two core problems of deliberative decision-making (its temporal and cognitive demands). Our results suggest that installing a continuous citizen group might be beneficial for the site-selection process. However, we are convinced that a consequent and elaborated selection process, as described here, is a necessary prerequisite to end up with a collaborative and efficiently working group.

Another significant factor is group cohesion, as mentioned by several CWG members. Those who remained active in the CWG reported the positive mood within the group, acknowledging that their open way of accepting one another helped them stick together for the whole project duration and accomplish their tasks. Tarpy et al. (2024, p. 35) distinguish two components of cohesion in groups. “Social cohesion refers to the degree to which group members are attracted to the group due to positive social relationships with other members.” This is clear when referring to the frequently mentioned positive social relationships among the CWG members. Social cohesion is influenced by regular in-person meetings. The other component—task cohesion—is closely related to “group characteristics such as being relatively small, having a formal leadership structure and a clear group goal” (Tarpy et al. 2024, p. 35). A review of the process indicates that two group members’ appointment to leadership positions and the group size are advantageous factors, as well as the predominantly clear goals for the group tasks. In conclusion, we propose that multiple processes and enabling factors led to the high trust values we measured. In particular, the wrap-up interview results suggest the line of reasoning presented in the next paragraph.

A selection process that looks for similar values among group members (Siegrist et al. 2000) ensures like-mindedness and high initial trust. Regular meetings and opportunities for informal face-to-face interactions increase familiarity, in turn strengthening social cohesion among group members and between the group and the researchers. Familiarity and the repeated experience that they can build mutual trust by getting to know each other on a more personal level adds personal/social trust to the general confidence in science. Meeting at eye level and being open signal the researchers’ genuine interest in the CWG’s knowledge and opinions. In terms of task cohesion, the group leaders are accepted, the goals set by the researchers are mostly clear, and the group size (15 ± 2) is favorable.

Some caveats or limitations should be mentioned, too. The approach described here is quite comprehensive and costly, in terms of both money and time. It requires good planning and budgeting in advance (Wong-Parodi 2022). Moreover, the time needed for workshop preparation and actual work together with the group may be a challenge for some researchers.

It has also become clear that not all processes can be controlled. One can establish certain basic conditions that may motivate citizens to participate in workshops and stay active for years. However, as participation in the workshops was voluntary, some were put off by the pandemic conditions; others could not always make the sometimes long journeys and stays. Additionally, the group members did not perceive themselves only as individuals who came together for scientific workshops. In some cases, personal contacts and friendships were formed that would last beyond the existence of the CWG in the project. Thus, there are processes beyond the control of the research team, which is not a problem per se, and it was not in our case. Nevertheless, even when a like-minded group like ours is recruited, dropouts can be expected. The members probably need to experience a (critical) number of common events and feelings of success to build this coherence. If there are negative side effects of too much coherence and intimacy with the researchers, these can also become problematic. In presenting our work with the CWG at various events, a recurring question has been whether the group is still critical enough to do its job, that is, to challenge the researchers. This dynamic needs further investigation. For now, one can be cautious about the changing role (or function) of such a group if its cohesion crosses a certain threshold and coherence becomes dysfunctional. However, as it gains additional knowledge, the group may develop a new function to facilitate the framing of new research questions and ideas. The approach may well be transferable to other environmental topics such as biodiversity, ecosystem services, or complex socio-ecological systems.

Regarding the impact of the project, the CWG members may be able to describe in an interview how their own views, perceptions, and behavior have changed (especially the empowerment point), but they are probably less able to assess what is changing in science and policy. Thus, their expectations concerning the impacts of the project results on the site-selection procedure and the disposal process are not as enthusiastic. Perhaps they are simply realistic. At the kick-off meeting, where the CWG members and the researchers first met, it was made clear that the project was not about applied research but application-oriented basic research and that the scientific results would unlikely have a direct impact on the political site-selection procedure. However, it took some time for all members to internalize this constraint. Transparency about the actual impact on decision-making processes is key.

We also identify potential limitations of the presented approach regarding transferability and generalizability. Further collaborations similar to the one discussed here are necessary to establish a proof of concept. Questions remain, such as whether the approach would be effective with individuals who are strongly opposed to nuclear waste and/or disposal, as well as how trust could be built in such cases. It is important to consider the roles of time and personal exchange in addressing these questions.

The cooperation of different individuals under artificially created framework conditions is subject to a whole range of (social) psychological variables and influencing factors (e.g., group dynamics), which make it fundamentally difficult to generalize. For certain forms of interaction (e.g., whether people are on first-name terms), a suitable procedure must certainly be agreed on for each specific project.

## Conclusion

What are the challenges and possible ways forward to make the approach of cooperating with citizens in a research project valuable? A range of issues should be addressed, including adequate funding, further conceptual development of the approach itself, the group’s potential professional bias, and options for institutionalization, multiplication, and application in other fields.

The applied psychological concepts worked well and helped in selecting the target group members, but these do not constitute a proof of concept yet. Potential evidence would need to be developed further, such as by selecting other, more diverse, or critical groups. Is it possible to generalize this method to other topics that trigger controversial views and problems in communicating scientific knowledge? To validate the approach, a systematic evaluation across many cases would be required. Securing funding for and coordinating such an endeavor present a significant challenge in scientifically proving the concept.

Another lesson pertains to the value of social or interpersonal processes among group members. Not all group dynamics and personal circumstances can be foreseen, such as likes and dislikes between individual members, illnesses, job changes, or general life plans of younger participants. This issue may be more relevant in our case because of the long duration of the collaboration.

Moreover, the CWG approach as chosen here could be a new way of “science communication”—not by communicating *to* or *through* citizens (as multipliers) but *with* them during a long-term collaboration. Researchers could observe how mental models and opinions change—on both sides, citizens and scientists—and thus adapt the mode of science itself accordingly. In terms of practical significance, our approach could provide a model for the interplay among the public disposal procedure (siting process), societal involvement, and scientific support.

To conclude, we therefore speculate whether this kind of collaboration is a way for the implementer of an HLW repository in Germany to work with the public to gain more understanding and trust. Policymakers and stakeholders could perceive the CWG approach as offering an “open and critical safe space” for the responsible administrators and the implementer to discuss and test ideas and strategies for the regional conferences where citizens from the affected municipalities would be invited to participate in the remaining steps of the site-selection procedure.

## Supplementary Information

Below is the link to the electronic supplementary material.

Supplementary file1 (DOCX 56 kb)

## Footnotes

1 See https://naturalsciences.ch/co-producing-knowledge-explained/methods/td-net_toolbox, 01/28/2025.

2 (10.25835/iuv28jg9).
