# Supplementary material for: A transdisciplinary approach to nuclear waste management: Opening research with a Citizens’ Working Group
Source: Ambio. 2025 Jul 16;55(2):385–401. doi: 10.1007/s13280-025-02214-9 (PMC12779802; doi:10.1007/s13280-025-02214-9)
Supplement: Supplementary file 1 — Supplementary file1 (DOCX 56 kb) [file 13280_2025_2214_MOESM1_ESM.pdf]

A transdisciplinary approach to nuclear waste management: opening research with a Citizens' Working Group

Authors: Roman Seidl[1,4*], Cord Drögemüller[1,2], Pius Krütli[3], Clemens Walther[1]

[1] Institute of Radioecology and Radiation Protection, Leibniz University Hannover, Hannover, Germany;

[2] Justus Liebig University Giessen, Giessen, Germany

[3] TdLab, ETH Zürich, Zurich, Switzerland

[4] Risk Dialogue Foundation, Zurich, Switzerland

***** Correspondence: roman.seidl@risiko-dialog.ch

Appendix 1: Survey on CWG Recruitment

Personal details

As we do not see your answers from the previous online survey, we will ask you demographic questions again. Please do not let this confuse you.

Your gender: Please select the appropriate category for you
female □ male □

What year were you born?
Year: _____

Socio-scientific / social / social participation

I am or was a member of a citizens' initiative (e.g. for environmental protection and nature conservation, against wind turbines).

□ Yes

□ No

If yes, in which area?

________________ [text box]

Do you deal directly with other people in your professional or charitable/voluntary work (e.g. pastoral care, therapy, teaching, youth work)?

- ☐ Yes
- ☐ No

How do you rate yourself personally?

Please indicate how much the statements below apply to you

	**applies fully**	**applies almost fully**	**applies to a great extent**	**applies partly**	**applies to some extent**	**does not apply**	**does not apply at all**
I am fundamentally interested in social and political decision-making processes.	☐	☐	☐	☐	☐	☐	☐
I want to understand the connections between technical possibilities, social requirements and political feasibility	☐	☐	☐	☐	☐	☐	☐
I am a broadly interested person	☐	☐	☐	☐	☐	☐	☐

Technical understanding

The following statements address your very personal attitude towards and use of modern technology. This is not about a single device, but about your attitude and your experience of using modern technologies in general.

Please indicate to what extent the following statements apply to you.

	**applies fully**	**applies almost fully**	**applies to a great extent**	**applies partly**	**applies to some extent**
I find it difficult to deal with new technology - I just can't do it most of the time.	☐	☐	☐	☐	☐
It is up to me whether I succeed in using new technical developments - it has little to do with chance or luck.	☐	☐	☐	☐	☐
I am always interested in using the latest technical equipment.	☐	☐	☐	☐	☐
I quickly take a liking to new technical developments.	☐	☐	☐	☐	☐

	**applies fully**	**applies almost fully**	**applies to a great extent**	**applies partly**	**applies to some extent**	**does not apply**	**does not apply at all**

In our society, rationality should be the determining factor in decision-making.	☐	☐	☐	☐	☐	☐	☐
The advantages of a highly technologized society compared to all other alternatives are obvious.	☐	☐	☐	☐	☐	☐	☐
In our society, decision-making should be characterized by humanity and be as participatory as possible.	☐	☐	☐	☐	☐	☐	☐

Experience with group work

How often are you involved in the following activities in your work?

	never	rare	occasionally	often	always
Guiding, training or teaching people, individually or in groups	☐	☐	☐	☐	☐
Giving speeches or presentations to at least five people	☐	☐	☐	☐	☐
Negotiating with other people inside or outside your company, firm or organization	☐	☐	☐	☐	☐

Ability to work in a team / personality

Please indicate to what extent the following statements apply to you.

[Compatibility]	**applies fully**	**applies almost fully**	**applies to a great extent**	**applies partly**	**applies to some extent**	**does not apply**	**does not apply at all**
I would rather work with others than compete with them	☐	☐	☐	☐	☐	☐	☐
I am better than most people and I know that too	☐	☐	☐	☐	☐	☐	☐
Human needs should always take precedence over economic considerations	☐	☐	☐	☐	☐	☐	☐

[Extraversion]	**applies fully**	**applies almost fully**	**applies to a great extent**	**applies partly**	**applies to some extent**	**does not apply**	**does not apply at all**
I am known as a warm and friendly person	☐	☐	☐	☐	☐	☐	☐
I like having lots of people around me	☐	☐	☐	☐	☐	☐	☐
I am dominant, self-confident and assertive	☐	☐	☐	☐	☐	☐	☐
I often feel like I'm bubbling over with energy	☐	☐	☐	☐	☐	☐	☐

We present various statements below.

Please indicate how true you find the statements when you compare them with yourself.

[Schwartz values]	**applies fully**	**applies almost fully**	**applies to a great extent**	**applies partly**	**applies to some extent**	**does not apply**	**does not apply at all**
I think it is important that all people in the world should be treated equally. I believe that everyone should have equal opportunities in life.	◻	◻	◻	◻	◻	◻	◻
It is important to me to listen to people who are different from me. Even if I disagree with them, I still want to understand them.	◻	◻	◻	◻	◻	◻	◻
It is important to me that others respect me. I want people to do what I say.	◻	◻	◻	◻	◻	◻	◻
It's important to me to develop new ideas and be creative. I like to do things in my own original way	◻	◻	◻	◻	◻	◻	◻

How do you rate yourself personally: Are you generally a person who is **very impatient** or a person who is **very patient?** Please answer on this scale...."

**very impatient**						**Very patient**
◻	◻	◻	◻	◻	◻	◻

Further social skills

Below you are asked to assess your own social skills. Please rate the extent to which the statements apply to you personally at this point in your training.

**[Ability to cooperate]**	**fully applicable**	**applicable**	**rather applicable**	**applicable to a medium extent**	**rather not applicable**	**not applicable**	**Not applicable at all**
Adhere to rules when dealing with others	◻	◻	◻	◻	◻	◻	◻
work together with others depending on the situation	◻	◻	◻	◻	◻	◻	◻

**[Ability to deal with conflict]**	**fully applicable**	**applicable**	**rather applicable**	**applicable to a medium extent**	**rather not applicable**	**not applicable**	**Not applicable at all**
Express constructive criticism	◻	◻	◻	◻	◻	◻	◻
seek a compromise in the event of differences of opinion	◻	◻	◻	◻	◻	◻	◻
Accept constructive criticism	◻	◻	◻	◻	◻	◻	◻

[Communication skills]	fully applica ble	applica ble	rather applica ble	applica ble to a medium extent	rather not applica ble	not applica ble	Not applica ble at all
express yourself clearly and precisely	◻	◻	◻	◻	◻	◻	◻
be able to listen to others	◻	◻	◻	◻	◻	◻	◻
discuss different views in an objective manner	◻	◻	◻	◻	◻	◻	◻

Time availability

The CWG will remain in existence for approximately 4 years after its constitution. However, we anticipate that the CWG will change within this period as members leave and join.

For how long do you expect to be able to work continuously in the CWG (about 2-3 meetings per year in the greater Hanover area)?
Please mark one of the periods mentioned according to your possibilities.

- up to 6 months
- 6-12 months
- 12-24 months
- longer than 24 months

Motivation and contribution

Finally, we ask you to briefly explain in a few sentences why you would like to work in the CWG and what you would like to/can specifically contribute to the work of the CWG and *The Project*.

_______________________________ [text box]

Thank you very much for your answers! The survey is now closed.

Appendix 2: Interview Guidelines

(English translation by the authors, project name replaced by “The Project”)

Interview – Number and Name:

Name evaluator:

Questions about your personal background (5 - 7 minutes)

- Perhaps you could briefly introduce yourself (Who are you? What is your current profession?)
- What did you spontaneously think when you completed the first survey on the subject of radioactive waste in Germany?
- What characterizes you as a person?
- How do you normally deal with dissent or conflict?

General information on the topic of final disposal (5 minutes)

- How much do you know about the current procedure for the final disposal of radioactive waste? What exactly excites you about the topic?
- Do you personally hope to gain something from your involvement in *The Project*?
- In your opinion, what is needed to deal with the topic "appropriately"?

Questions about *The Project* and its progress (7 - 10 minutes)

2-3 sentences on the work of the CWG, as we intend it to be

- Perhaps you have read up on the *The Project* homepage or even looked at the project description. What do you know about *The Project*?
- What do you expect from the work in the CWG or how do you imagine this work, formally, in terms of content, humanly?
- What do you expect from the interaction with the scientists in *the project*?
- The CWG should be constituted before fall if possible.
    - What do you think of personal meetings with the other CWG members?
    - Would you be prepared to hold this as a video conference if necessary?
- What is your availability this year and beyond?

Questions from the person to *the project* (5 - 7 minutes)

- What questions do you have about *the project* or the process?
- Are there certain conditions for you to take part in the CWG? Or what are the "no-goes"?
- Are there any questions from your side that we should/must clarify?

Appendix 3: Observation Matrix

**Observer abbreviation:**	xy
**Date:**	October 21, 2022 Friday
**Type of meeting:**	
**Objective of meeting:**	Observation of trust/mistrust
**Participants:**	

Note: The exact time can be added by simply pressing Ctrl + Shift + .

			**CONTENT / VERBAL**					
**Event**	**Time**	**Who**	**Type of communication**	**Topic/content**	**Indicators (trust/mistrust)**	**Quotes, notes, especially on the subject of trust**	**NONVERBAL**	

**Abbreviation of the indicators**					
**Type of communicati**		**Indicators trust / mistrust**		**Nonverbal indicators**	
AN	Answer	AP	Approval	SH	Shake head
DI	Discussion	CS	Changing the subject	No	Nodding
DI	Discussion lead	CO	Concealment	Sm	Smiling
EX	Explanation	CON	Contradiction/criticism		
IN	Input (e.g. technical)	CT	Control (question)		
MO	Moderation/	CR	Critical		
PR	Presentation	DI	Distortion		
QUI	Query to individual	FA	Factual/objective		
QA	Question to all	OH	Offer/ask for help		
QI	Question to individual	DC	Passing on/disclosing critical (confidential or insider) information		
RM	Remark	PR	Provocation/aggression		
		RE	Rejection		
		CO	Seeking contact with certain people		
		SI	Show interest		
		UE	Undermining expertise		

Appendix 4: Survey on attitudes after workshop [examples]

(English translation by the authors)

How satisfied are you with the project meeting?
Please indicate to what extent the following aspects apply to your involvement in the project.

	**fully applica ble**	**applica ble**	**rather applica ble**	**applica ble to a mediu m extent**	**rather not applica ble**	**not applica ble**	**Not applica ble at all**
There was an atmosphere of openness	☐	☐	☐	☐	☐	☐	☐
There is a climate of mutual trust between AGBe members	☐	☐	☐	☐	☐	☐	☐
The event was conducted professionally	☐	☐	☐	☐	☐	☐	☐
I was encouraged to reflect or think further	☐	☐	☐	☐	☐	☐	☐

Please indicate to what extent the statements currently apply to you.

	**fully applica ble**	**applica ble**	**rather applica ble**	**applica ble to a mediu m extent**	**rather not applica ble**	**not applica ble**	**Not applica ble at all**
I trust the researchers in the project	☐	☐	☐	☐	☐	☐	☐
Researchers in the project are professionally competent	☐	☐	☐	☐	☐	☐	☐
Researchers in the project take participation seriously	☐	☐	☐	☐	☐	☐	☐
the project researchers have similar values to mine	☐	☐	☐	☐	☐	☐	☐
All in all, I feel comfortable as part of the project	☐	☐	☐	☐	☐	☐	☐

Please indicate to what extent the following aspects apply to your involvement in the project.

	**fully applica ble**	**applica ble**	**rather applica ble**	**applica ble to a mediu m extent**	**rather not applica ble**	**not applica ble**	**Not applica ble at all**
Fairness: I expect everyone involved to treat each other fairly/respectfully	☐	☐	☐	☐	☐	☐	☐
Transparency: All relevant topics must be disclosed and discussed	☐	☐	☐	☐	☐	☐	☐

Decision-making and procedural rules are defined jointly	☐	☐	☐	☐	☐	☐	☐
Reliable expectations: The role and influence of the AGBe are clearly regulated	☐	☐	☐	☐	☐	☐	☐

Question: Now three questions on the possibility of retrieving radioactive waste.
Please indicate to what extent the following statements apply in your opinion.

	**fully applica ble**	**applica ble**	**rather applica ble**	**applica ble to a mediu m extent**	**rather not applica ble**	**not applica ble**	**Not applica ble at all**
I am generally against the possibility of retrieving radioactive waste	☐	☐	☐	☐	☐	☐	☐
The possibility of correcting errors by retrieving the waste creates trust	☐	☐	☐	☐	☐	☐	☐
Retrieving the waste involves risks	☐	☐	☐	☐	☐	☐	☐

Appendix 5: Interview guideline for the CWG-wrap up interviews

(English translation by the authors)

Interviewer:

Interviewee (abbreviation): Int_01

Date:

**No.**	**Topic**	**Question**
**1**	**Motivation**	Active participants: Has the motivation to participate in the project (especially in the workshops) changed over time?<br>If yes - how does this manifest itself or has it manifested itself?<br>Inactive: Has the motivation for participating in the project (especially in the workshops) changed over time? Why are you no longer actively involved?
	NOTES…..	
**2**	**Knowledge**	Name the most important point from your perspective [most significant change]: What have you learned or "taught" others, or what do you think you have been able to contribute to?<br>What findings from the CWG work could be important for the further disposal process
	NOTES…..	

3	**Trust**	What were the key points for the development of trust from the perspective of the CWG: Mutual (within the CWG) and towards the scientists in the project<br><br>How did trust in the scientists develop and how has it developed over time (compared to before the start and now)?<br>[if not already mentioned] Was trust also a reason for "staying in" or "staying away"?
	NOTES…..	
4	**Science**	Has the collaboration made the contributions of the respective research areas and disciplines clearer to you? [That is: as it become clearer what the individual people are doing and what it is good for?]<br><br>Where are there currently still uncertainties, or what would be needed for an even better understanding of the topic?<br><br>How relevant do you think the content developed by the project is? Will they be well received in society?
	NOTES…..	
5	**Monitoring**	What results or experiences were particularly memorable after the three workshops on monitoring?
	NOTES…..	
6	**Host rock**	After your involvement with final disposal, which host rock do you consider most suitable and why?<br><br>Why might the option of retrieving waste make sense despite the technical/social complexity involved?
	NOTES…..	
7	**Understanding of roles**	From your point of view, what was your role in the project and were you able to fulfill it?<br><br>Did your view of your own role in the project change?<br><br>[Or that one expects scientific knowledge contributions from participants of the CWG;<br>or see yourself more as *an extended peer group*]
	NOTES…..	
8	**Personal conclusion...**	Finally...<br><br>What has the insight into research brought you personally?
	NOTES…..	

Appendix 6: Summarized results from the wrap-up interviews

Table A 1: Shows summarized results for each category.

Category: Trust	Total	Summary
Basic trust in science	9	Nine interviewees explained their basic trust in science.<br>Of these, 3 interviewees linked this to their own background.<br>Two interviewees are of the opinion that trust has grown (without giving specific reasons or due to the competence of researchers in the subject matter and its communication). Three respondents also mentioned the personal component, which strengthened their trust in science.
Group cohesion / safe space	7	Group feeling and cohesion is mentioned by 5 interviewees in connection with building trust. One describes the group as a safe space.
Openness / transparency / honesty	9	The open and honest nature of the researchers, which generated trust (limits of knowledge, various questions within and outside the topic, welcome as a layperson), was mentioned by 7 participants.<br>In addition, the open interaction with each other in the group was mentioned by 3 as appreciated and promoting trust.<br>One participant suspected that this helped not only the CWG but also younger researchers to open up.
Eye level / respectful interaction (with each other and with researchers)	5	Appreciative interaction with each other (2 times), meeting at eye level (2 times) and the experience of being taken seriously (2 times) were mentioned as trust-promoting. One interviewee said that the visit of a scientist to the regulars' table, who took the extra time to give a scientific input, was trust-building.
Getting to know each other and researchers	7	With regard to researchers, 4 participants said that they had gained trust through the personal relationship (being more tangible, being also “just people”, feeling a personal connection).<br>For the growing trust within the group (among the CWG and researchers), getting to know each other personally (regular exchange, sharing of personal topics) was mentioned by 4 interviewees as an important factor.
Getting to know the project	1	One P. described how she was unclear at the beginning about how the project would work and what to expect. Once it was clear to her that the project was scientific, neutral and serious, her trust in science was reaffirmed.
Joint activities (regulars' table, meals, etc.)	3	Private life is also shared, e.g. going out to dinner together in the evening. For 1 interviewee this has strengthened trust. The other segments could be indirectly linked to trust (in the sense of group cohesion). For example, seeing each other more than previously planned (monthly at the regulars' table) or the whole group going out for dinner.
WhatsApp messages / group	1	One interviewee mentioned WhatsApp messages and the group as valuable for keeping in touch, knowing where the others were (when absent from meetings, for example). She also mentioned that the group made an effort to keep in touch.
Working basis	2	Coming together in Hanover was perceived as positive by 2 interviewees. One interviewee says that a “solid basis was created” by discussing the idea of the project. The other interviewee mentions the working basis as something positive.
Be on first name terms	1	For 1 interviewee, the scientists being on first-name terms was something positive.

Interdisciplinarity	1	The involvement of various disciplines was cited by 1 interviewee as trust-building, as it meant that arguments were (counter)argued from different perspectives.
SUM	46	

**Mistrust**	**Total**	**Summary**
Conflict (among each other)	1	Two participants stated that discomfort in the group or with several people in the group could have led to a termination.
Lack of communication (among each other)	1	One interviewee expressed the wish that group members should communicate their (longer-term) absence (individual case).
Discomfort in group	2	For one participant to stop, there would have had to be a major conflict that would have destroyed trust.
Not taken seriously (by researchers)	1	Not being taken seriously by researchers would have been part of the reason for another group member to leave the project.
Scam skepticism	1	One interviewee was initially suspicious as to whether it was a scam, a way of obtaining email addresses.
SUM	6	

Note: This category includes more counterfactual issues, in the sense that if this or that had happened, it would have decreased trust. One concern was factual, however, as two women quietly left the group (independently), which was not appreciated by the rest of the members. They would have expected an open explanation.

**Motivation**	**Total**	**Summary**
At the beginning	***25***	
Problem solving (making a contribution, responsibility, involvement)	10	Six participants stated that they want to get involved, collaborate or be involved in this process. Three named the topic of radioactive waste and its disposal as an important topic (feel responsible, entire society affected). One interviewee expressed a motivation to pass on what they had learned (as a multiplier) in local participation or to their personal contacts.
Interest in the topic (general, project)	8	Six interviewees expressed interest in the topic in general. Three were (also) interested in the project (interdisciplinarity, project procedure, curious “what is it?”)
Thirst for knowledge / willingness to learn	4	Getting more specific information was mentioned by 3 interviewees as a partial motivation. One interviewee named curiosity (regarding the project) and thirst for knowledge as motivation to participate.
Topic of final disposal / radioactive waste	2	Two participants said that they found radioactive waste exciting as a topic, with 1 P. also emphasizing her interest in interim storage and its current topicality.
Social contacts / exchange	1	One of the motivations for one interviewee to take part was to come into contact with a diverse group (backgrounds, ages) and to work together.
Changed / new during the course of the project	***20***	
Social contacts / exchange	8	Three interviewees mentioned that the group was a motivation to stay in it (harmonized, grown together, close connection, etc.). The mutual exchange was mentioned by 4 interviewees as a motivation for various reasons, as progress was seen, exciting topics addressed (also outside of the project) or positive experiences were made at meetings.

Problem solving (making a contribution, responsibility, involvement)	4	Two interviewees find the project important and see an urgency and added value (since knowledge acquisition has increased, science communication). One participant is more motivated to be involved, but is also worried because he feels that processes are "off track" in terms of communication. However, not within the group (CWG & researchers), but in terms of the political process.
Thirst for knowledge / willingness to learn	4	As their knowledge increased over the course of the project, 2 interviewees felt more curious and inspired by the new areas that opened up to them. Two also cited the opportunity to acquire new knowledge as a motivation to stay involved. Knowledge within and outside the subject matter was mentioned, as well as methodological approaches.
Interest in the topic (broader: environmental pollution, transdisciplinary project)	2	Interest in various disciplines involved has grown for one participant, while another has discovered new interests through interdisciplinary cooperation.
Topic of final disposal / radioactive waste	1	One participant cited the current world situation, including the war in Ukraine, as one reason why the topic of final disposal and radioactive waste has become more important (for the participant personally).
Change of location, travel	1	The change of location (e.g. universities in different cities) was seen by one interviewee as exciting and motivating for participation.
SUM	45	

**Role**	**Total**	**Summary**
Role change	***63***	
Layperson / outside perspective	8	Role as an outsider (observer, standing on the sidelines) was described by 5 participants. Three of them explained that they did not have much knowledge on the topic/are laypersons. In addition, 3 interviewees did not ascribe themselves an individual role, but placed the group at the center (part of a group).
Informed / Involved layperson	5	A change in role to informed layperson with increasing expertise (involvement, information transfer) was mentioned by 8 participants. Six interviewees said that over time they contributed their own input (knowledge, experience, opinions, discussion points, perspective) in the exchange between CWG and researchers. A mediating role between society and science was described by 2 participants (translation and mediation of understanding between different language codes).
Mediator role	4	
Role change due to more knowledge / involvement	3	
Contribute own input	10	
Representation of society / diversity within CWG	2	Role as group members, which is generally diverse in order to represent society (diversity in age, affectedness, gender, profession) was mentioned by 4 participants.<br>On the other hand, one participant said that it was difficult for employees to participate (vacation days limited) and that mainly non-employed people (students, pensioners, house "persons") are eligible for the project.
Representative of society / diversity in CWG	8	
Not all of society covered	6	

Complexity of the topic	6	From six participants mentioning this issue, four explained that they recognized the complexity of the topic in the course of the project (difficult to break down, connections, simplified prejudices). One also said that in a complex topic it does not make sense for their own role in the CWG to acquire in-depth knowledge, but rather to understand the big picture and the whole.
Contribution to science	***45***	
Comprehensible science communication / dialog with the population at eye level	8	Seven participants saw a contribution of the CWG to science with regard to comprehensibility in science communication (between researchers and the public; low-threshold language, understandable for all, adapt language). Three respondents also mentioned dealing with lay people / the public on an equal footing as a positive learning experience of the project.
Suggestions / change of perspective through exchange with CWG	4	Four participants explained that CWG made a contribution to science by participating in the exchange and bringing in a different perspective (own discussion points, opinions, feedback, priorities)
Moderation techniques / stimulating formats	3	The experience-oriented transfer of knowledge and the testing of moderation techniques and tools was seen by 3 interviewees as a contribution to science.
No scientific contribution from CWG	3	Three participants explicitly stated that the CWG could not make a scientific (that is, technical) contribution (time limit, no scientists, not their task).
Contribution to politics	4	Three interviewees hope that the experiences from the project (interaction with the public at eye level, bringing the topic closer to the public) will be echoed in politics (steering the site selection procedure). For one interviewee, the policy maker asked whether the project is relevant for further disposal procedures and possibly the contribution of CWG for de-escalating an emotional debate.
Contribution to determining the location of the DGR	2	Two participants thought at the beginning of the project that site selection was the project objective. They then realized during the course of the project that this was not the case (but independent research).
Contribution of CWG unclear	2	Two participants expressed uncertainty as to whether the project could contribute something for others.<br>One participant sees possible indirect benefits (guidance & support of processes, advice).
Change in attitude / formative: Monitoring	10	For 10 participants the realization that monitoring also entails a loss of safety was formative. At the beginning, they were of the opinion that as much monitoring as possible (trust, safety), then a trade-off with the loss of safety (drilling, instability). One also mentioned the energy consumption of monitoring as a new insight, and one also saw the trade-off on the background of ever-improving technology.
Change in perception: Radioactivity / nuclear waste	2	Two participants described that delving deeper into the topic / contact with experts took away their fears about radioactivity.
Change of attitude / formative: Retrievability	2	One interviewee, as with monitoring, was initially convinced of retrievability. With more in-depth insight and exchange with researchers on the associated risks, grew unsure of their own opinion. An important realization for one participant was that the repository does not have to be final, certain situations can force retrieval.
Change of attitude / formative: Host rocks	5	New knowledge of the role of host rock was important for five interviewees. Two described the role of the container as rather

		secondary compared to the host rocks, which was an important insight for them. Some respondents mentioned their own lack of understanding of various aspects relating to host rocks.
Multiplier / networking	***11***	
Public relations	7	At the beginning, according to 2 participants, public relations work was often discussed. Two mentioned the possibility of holding workshops at schools, while 3 mentioned that the timing plays an important role (not now; later, when the disposal site is identified). When the location is in place, the CWG could be confidence-building according to 2 interviewees.
Interest from other stakeholders	2	Two participants mentioned that they had noticed interest from official actors in their work and role.
Attending event privately	2	One interviewee said that she attends lecture series also privately as her involvement in the topic progresses. One also attended an project event privately (not as CWG-member) on their own costs.
Empowerment	***34***	
Knowledge acquired	10	10 participants commented positively on the fact that they were able to acquire a lot of new knowledge/understanding on the topic (personally gained a lot; did not otherwise deal with the topic; privilege to take part). For one this also came with the realization that she is still receptive, capable of learning and it strengthened her/his self-confidence. One interviewee also said that she had learned a lot about transdisciplinary research and formats.
Exchange with others (AGB and researchers)	5	Positive experiences of working with a mixed group were mentioned by 2 participants. One mentioned the strengthening of her self-confidence through the collaboration with the scientists. 3 also mentioned that they got to know different people and see this as personal enrichment.
Questioning skills / eloquence / discussion culture	4	Three participants mentioned the good discussion culture and that they appreciated and learned a lot from it (sharing personal opinions without being aggressive, avoiding negative reactions from others). Sharing opinions without aggressive, negative reactions from others, avoiding dominance by a few).
Disempowerment: Dominance in exchange	1	One participant mentioned that she perceived dominance by a few when workshop discussions were not moderated. Online events were therefore easier for her (as one can raise the virtual hand). She was nevertheless able to strengthen her eloquence through the exchange with researchers.
Writing a report	5	Five participants described the CWG's involvement in the report and anthology as positive (helpful for studies, fun to write, unusual opportunity, being taken seriously).
Giving a lecture, workshop	1	One interviewee mentioned taking part in various events and workshops. Self-confidence has grown through the request for input in public (“not as stupid as I thought”) and the appearance has become more uninhibited (also through more knowledge).
Broadening horizons	3	Insight into research work in general was mentioned (1), as was insight into another discipline adding to the own background (2).
Moderation techniques	2	One participant noted that she had learned a lot about various facilitation techniques (World Café, designing workshops, etc.). She also learned a lot about presentation techniques (World Café, workshop design, etc.) and how to present herself (facial expressions, body language). Another was able to better absorb information and give her own input thanks to the structure of

		the meetings (reading, listening to a presentation, then discussing in small groups).
Interest in collaboration (from researchers)	2	One participant recognized genuine interest on the part of researchers right from the start, which was enormous empowerment. Another found it beneficial that researchers approached her with opportunities to contribute knowledge, "raised her to eye level".
Speaker function	1	One participant mentioned gaining experience in the role of (one of the) the group's speaker(s).
SUM	153	
